# Catalytic-dependent and -independent roles of TET3 in the regulation of specific genetic programs during neuroectoderm specification

**DOI:** 10.1038/s42003-024-06120-w

**Published:** 2024-04-05

**Authors:** Harmony C. Ketchum, Masako Suzuki, Meelad M. Dawlaty

**Affiliations:** 1 Ruth L. and David S. Gottesman Institute for Stem Cell and Regenerative Medicine Research, Albert Einstein College of Medicine, Bronx, New York, USA; 2 Department of Genetics, Albert Einstein College of Medicine, Bronx, New York, USA; 3 Department of Developmental & Molecular Biology, Albert Einstein College of Medicine, Bronx, New York, USA; 4 Department of Nutrition, Texas A&M University, College Station, Texas USA

**Keywords:** Stem-cell differentiation, Embryonic stem cells, DNA methylation

## Abstract

The ten-eleven-translocation family of proteins (TET1/2/3) are epigenetic regulators of gene expression. They regulate genes by promoting DNA demethylation (i.e., catalytic activity) and by partnering with regulatory proteins (i.e., non-catalytic functions). Unlike *Tet1* and *Tet2*, *Tet3* is not expressed in mouse embryonic stem cells (ESCs) but is induced upon ESC differentiation. However, the significance of its dual roles in lineage specification is less defined. By generating TET3 catalytic-mutant (*Tet3*^*m/m*^) and knockout (*Tet3*^*–/–*^) mouse ESCs and differentiating them to neuroectoderm (NE), we identify distinct catalytic-dependent and independent roles of TET3 in NE specification. We find that the catalytic activity of TET3 is important for activation of neural genes while its non-catalytic functions are involved in suppressing mesodermal programs. Interestingly, the vast majority of differentially methylated regions (DMRs) in *Tet3*^*m/m*^ and *Tet3*^*–/–*^ NE cells are hypomethylated. The hypo-DMRs are associated to aberrantly upregulated genes while the hyper-DMRs are linked to downregulated neural genes. We find the maintenance methyltransferase *Dnmt1* as a direct target of TET3, which is downregulated in TET3-deficient NE cells and may contribute to the increased DNA hypomethylation. Our findings establish that the catalytic-dependent and -independent roles of TET3 have distinct contributions to NE specification with potential implications in development.

## Abstract (web summary)

Specification of neuroectoderm from TET3 enzymatic deficient and knockout embryonic stem cells reveals distinct catalytic-dependent and -independent roles of TET3 in activation of neural and suppression of mesodermal programs, respectively.

## Introduction

The ten-eleven translocation family of enzymes (TET1/2/3) are DNA dioxygenases that are essential for embryonic development. They catalyze DNA demethylation through successive oxidation of 5-methylcytosine (5mC) to 5-hydroxymethylcytosine (5hmC), 5-formylcytosine (5fC) and 5-carboxylcytosine (5caC)^1–3^. These bases can be excised by thymine DNA glycosylase (TDG) and removed through base excision repair pathways completing active DNA demethylation^3–5^. 5hmC is a stable base and is recognized by other proteins suggesting it may have independent regulatory roles^6^. It can evade recognition by UHRF1 and thus interfere with recruitment of DNMT1 to promote passive DNA demethylation during replication^6,7^.

TET enzymes are dynamically expressed during embryogenesis and play essential roles in gene regulation during development and in embryonic stem cell (ESC) biology^8,9^. *Tet1* and *Tet2* are highly expressed in ESCs, whereas *Tet3* is not detectable^10^. While *Tet1/2* levels decline during ESC differentiation, *Tet3* is induced^9,11^. Loss of TET1 or TET2 in ESCs does not block pluripotency but compromises expression of lineage specific genes and ESC differentiation^12–14^. TET1 and TET2 deficient mice are viable, whereas TET3 loss results in perinatal lethality with no overt developmental defects^13,15,16^. TET1/3 double knockout embryos are embryonic lethal by E10.5 with impaired forebrain development and increased transcriptome variability^17^. TET1/2/3 triple knockout (TKO) ESCs have severe differentiation defects consistent with TET TKO embryos failing to complete gastrulation and die by E8.5^10,16^. These finding support TET enzymes have both redundant and unique roles in embryogenesis.

*Tet3* is uniquely expressed in the zygote where it is required for promoting DNA demethylation of the paternal genome^16,18–21^. Deletion of TET3 in oocytes causes developmental defects and lethality by mid-gestation^16,22^. Unlike *Tet1/2*, *Tet3* is not expressed in the inner cell mass of the blastocyst nor in ESCs, but is induced during ESC differentiation and post-implantation development^11^. However, its roles in post-implantation development are less defined. TET3 has been shown to be important for development of some somatic cell types such as immune cells^23,24^, neural stem cells^11,25–27^ and cardiomyocytes^28^, and is also deregulated in some cancers^29,30^. TET3 mutations have also been implicated in human developmental disorders with all patients showing craniofacial abnormalities, and few with cardiovascular, motor, and speech deficits^31,32^. These studies underscore the need to understand the requirement of TET3 during development.

Over recent years, our lab and others have shown that TET enzymes can regulate gene expression independent of their enzymatic activity, which entails partnering with or recruitment of other transcription factors and epigenetic modifiers to chromatin^5,12,14,33–36^. Specifically, we and others have shown that in ESCs, TET1 recruits PRC2 and SIN3A to promoters of bivalent genes for H3K27 trimethylation and deacetylation, respectively^12,33,34,37^. Additionally, in ESCs, we have shown that TET2 recruits SIN3A to active enhancers of its target genes independent of its catalytic activity^14^. Further, in hematopoietic stem cells, TET2 non-catalytic functions are important for regulating the lymphoid lineage and preventing lymphoid malignancies^35^. The non-catalytic functions of TET3 are less defined and studies have been limited to lineage committed cell types. In immune cells, TET3 partnership with HDAC1 is critical for eliciting an immune response following viral infection^24^. Likewise, TET3 non-catalytic functions are important in astrocyte specification from neural stem cells (NSCs)^25,26^. However, the catalytic and non-catalytic functions of TET3 during early lineage specification have not been investigated. Here, we have used TET3 catalytic deficient and knockout mouse ESCs to study how TET3 regulates genes during ESC differentiation to neuroectoderm (NE), a lineage where *Tet3* is highly expressed. We have identified distinct catalytic dependent and independent roles of TET3 in activation of neural and suppression of mesodermal programs as ESCs commit to NE. These findings not only define the roles of TET3 in lineage specification but may also have potential implications in neurodevelopment where TET3 is mutated or dysregulated.

## Results

### Generation of TET3 catalytic mutant (*Tet3*^*m/m*^) and knockout (*Tet3*^*–/–*^) mouse ESCs and their differentiation to neuroectoderm (NE)

**Fig. 1:**
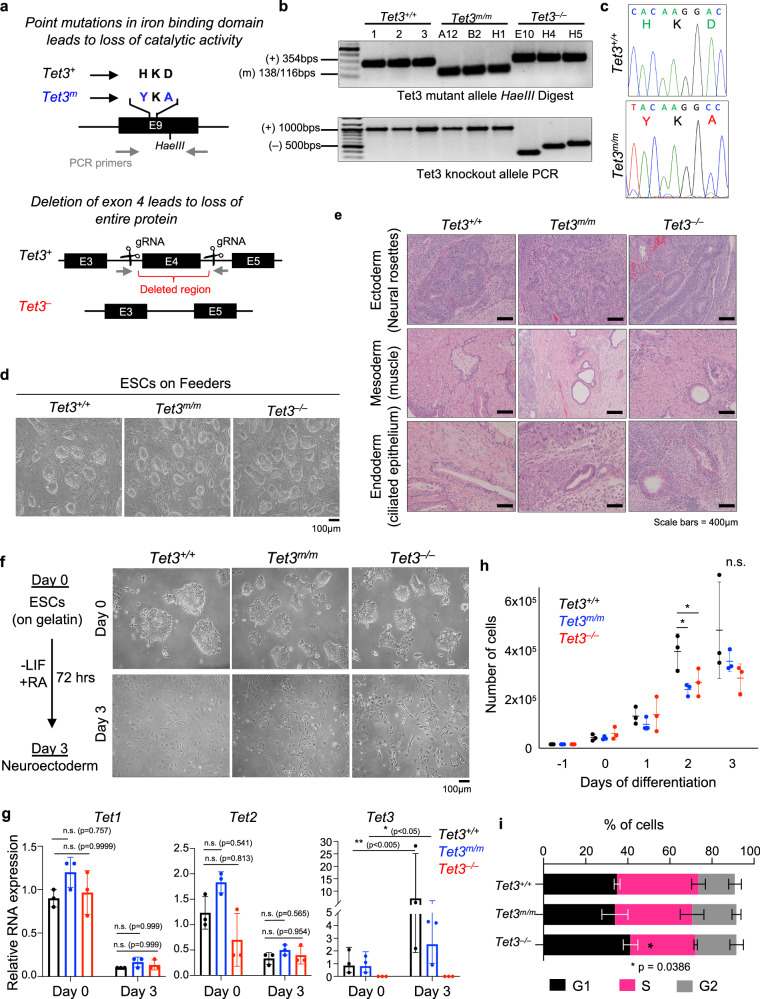
Generation of *Tet3*^*m/m*^ and *Tet3*^*–/–*^ mouse ESC and their differentiation to NE. **a** Schematic of the gene targeting strategy to generate catalytic mutant (*Tet3*^*m*^) and knockout (*Tet3*^*–*^) mouse ESCs. **b** Genotyping of *Tet3*^*m/m*^ clones by RFLP (restriction fragment length polymorphism) using *HaeIII* enzyme (top). Correctly targeted (mutated) allele bands are 138 bp and 116 bp. Allele without mutation is 354 bp. (3 independent clones were generated A12, B2, and H1). Genotyping of *Tet3*^*–/–*^ clones by PCR (bottom). Amplification of a shorter fragment (~500 bps) confirms deletion of exon 4 (3 independent clones were generated E10, H4, and H5). **c** Sanger sequencing confirming mutations for amino acid substitutions HKD to YKA in *Tet3*^*m/m*^ ESCs. **d** Representative brightfield images of ESCs cultured on feeder cells. Scale bar = 100μm. **e** Hematoxylin and Eosin (H&E) staining of sections of teratomas derived from ESCs of indicated genotypes. Scale bars = 400 μm. **f** Representative brightfield images of *Tet3*^*+/+*^*, Tet3*^*m/m*^, and *Tet3*^*–/–*^ ESCs on gelatin (top) and after differentiation to NE by LIF withdrawal and RA treatment for 72 h (bottom). Scale bar = 100 μm. **g** Quantification of *Tet1/2/3* mRNA levels at day 0 and 3 of –LIF + RA treatment. Data normalized to *Gapdh* expression. **h** Growth curve of ESCs differentiated to NE. Viable cells were counted during each day of differentiation. **i** Cell cycle analysis of NE cells at day 3 of –LIF + RA treatment. Note *Tet3*^*–/–*^, but not *Tet3*^*m/m*^, cells show a reduction of cells in S phase. For all panels error bars represent SD and * indicates statistical significance (*p* < 0.05).

To define the catalytic and non-catalytic functions of TET3, we generated TET3 catalytic deficient (*Tet3*^*m/m*^) and knockout (*Tet3*^*–/–*^) mouse ESCs using CRISPR/Cas9 mediated gene editing. To generate TET3 catalytic deficient ESCs, we used a guide RNA (gRNA) targeting exon 9 and a donor oligo coding for amino acid substitutions H950Y and D952A in the iron binding site of TET3 as well as a *HaeIII* restriction site to allow for screening of targeted clones by restriction fragment length polymorphism (RFLP) (Fig. 1a). The amino acids H950 and D952 are conserved among all TET enzymes and their respective substitutions to H950Y and D952A is shown to abrogate the catalytic activity of TET3^1,24,26,38^. To generate *Tet3*^*–/–*^ ESCs, two gRNA flanking exon 4 were used to delete the exon (Fig. 1a), which leads to an out of frame transcript with an immediate stop codon resulting in complete loss of TET3 as shown before^10^. Properly targeted *Tet3*^*m/m*^ ESC clones (*n* = 3) were validated by RFLP and Sanger sequencing (Fig. 1b, c) and deletion of exon 4 in *Tet3*^*–/–*^ ESC clones (*n* = 3) was confirmed by PCR (Fig. 1b). *Tet3*^*m/m*^ and *Tet3*^*–/–*^ ESCs maintained normal morphology in culture (Fig. 1d) and remained pluripotent forming teratomas with structures of the three germ layers (Fig. 1e) consistent with previous reports that loss of TET3 does not overtly effect pluripotency.

*Tet3* is not expressed in ESCs but is induced upon differentiation and is highly expressed in the neural lineage. Therefore, we differentiated *Tet3*^*+/+*^, *Tet3*^*m/m*^, and *Tet3*^*–/–*^ ESCs to neuroectoderm (NE) by LIF withdrawal and retinoic acid (RA) treatment for three days^39^. *Tet3*^*m/m*^ and *Tet3*^*–/–*^ NE cells were morphologically indistinguishable from their wildtype counterparts (Fig. 1f). Expression of *Tet3* mutant mRNA in *Tet3*^*m/m*^ and loss of exon 4 containing transcripts in *Tet3*^*–/–*^ NE cells were confirmed by RT-qPCR (Fig. 1g). We note that there was a reduction in mRNA levels of mutant *Tet3* suggesting some instability of this transcript. We also confirmed that *Tet1* and *Tet2* transcript levels were not affected in *Tet3*^*m/m*^ or *Tet3*^*–/–*^ ESCs, and both were downregulated upon ESC differentiation (Fig. 1g). While growth of *Tet3*^*m/m*^ or *Tet3*^*–/–*^ ESCs were unaffected, upon differentiation to NE, we found a subtle trend in reduced proliferation of NE cells which reached significance at day 2 (Fig. 1h). Cell cycle analysis of NE cells confirmed a mild increase in G1 and decrease in S phase cells in *Tet3*^*–/–*^, but not in *Tet3*^*m/m*^, cultures (Fig. 1i) suggesting that Tet3 non-catalytic functions may subtly influence cell cycle progression of NE cells. Since *Tet3*^*m/m*^ cells only lack the catalytic activity of TET3, their comparisons to *Tet3*^*–/–*^ cells allows for dissecting the catalytic and non-catalytic functions of TET3 in NE specification.

### TET3 promotes activation of neural genes and suppression of mesodermal genes through catalytic and non-catalytic functions, respectively

**Fig. 2:**
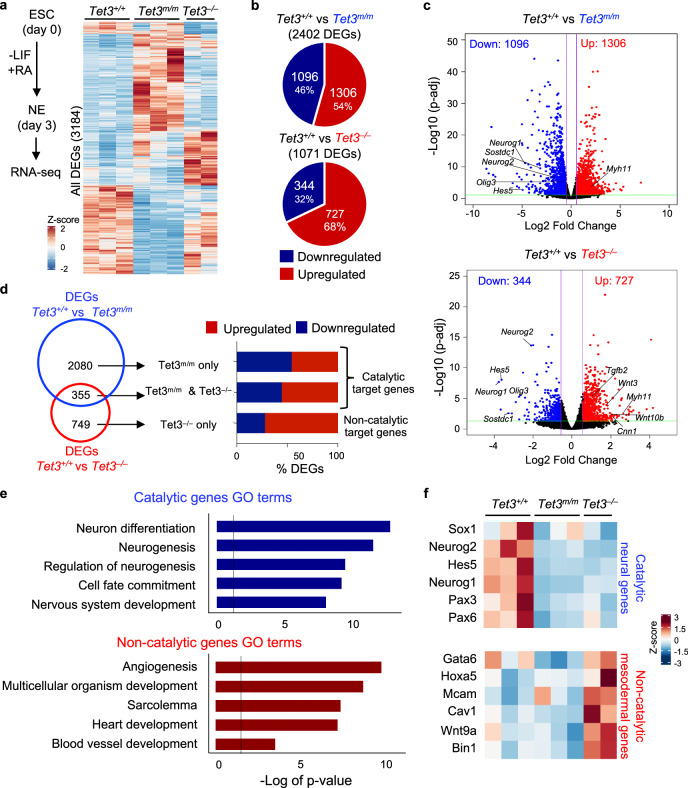
TET3 regulates activation of neural genes and suppression of mesodermal genes during differentiation of mouse ESCs to NE. **a** Heatmap of all 3184 differentially expressed genes (DEGs, fold changes > 1.5, FDR < 0.05) in *Tet3*^*m/m*^ and *Tet3*^*–/–*^ cells on day 3 of –LIF + RA. Color represents relative expression extracted from normalized counts. (Three independent *Tet3*^*+/+*^ and *Tet3*^*m/m*^ clones and two independent *Tet3*^*–/–*^ clones were used for this analysis). **b** Up and downregulated DEGs between *Tet3*^*+/+*^ vs *Tet3*^*m/m*^ (top) and *Tet3*^*+/+*^ vs *Tet3*^*–/–*^ (bottom). **c** Volcano plots showing DEGs between *Tet3*^*+/+*^ vs *Tet3*^*m/m*^ (top) and *Tet3*^*+/+*^ vs *Tet3*^*–/–*^ (bottom). Some downregulated neural genes and upregulated mesodermal genes are noted. **d** Venn diagram (left) showing the overlap of DEGs between *Tet3*^*m/m*^ and *Tet3*^*–/–*^. The percent of up and downregulated genes in each category is plotted (right). Genes deregulated in *Tet3*^*m/m*^ are catalytic targets of TET3. Genes uniquely deregulated in *Tet3*^*–/–*^are TET3 non-catalytic targets. **e** Gene Ontology (GO) analysis of all catalytic target genes (top) and non-catalytic target genes (bottom). Line represents significance (*p* < 0.05). **f** Heatmap of selected catalytic target neural genes that are downregulated in both *Tet3*^*m/m*^ and *Tet3*^*–/–*^ and selected non-catalytic target mesodermal genes uniquely upregulated in *Tet3*^*–/–*^ cells.

To identify catalytic and non-catalytic target genes of TET3, we compared the transcriptomes of *Tet3*^*+/+*^, *Tet3*^*m/m*^, and *Tet3*^*–/–*^ NE cells (harvested 72 h after LIF withdrawal and RA treatment) by RNAseq (Supplementary Fig. 1a). Sample clustering by Euclidean distance revealed that the biological replicates clustered with each other (Supplementary Fig. 1b), and principal component analysis (PCA) confirmed they were separated by genotype (Supplementary Fig. 1c). We identified a total of 3,184 differentially expressed genes (DEGs) between *Tet3*^*+/+*^, *Tet3*^*m/m*^, and *Tet3*^*–/–*^ cells showing distinct expression profiles in each genotype (Fig. 2a). The expression of *Tet1* and *Tet2* was not affected in *Tet3*^*m/m*^ and *Tet3*^*–/–*^ cells (Supplementary Fig. 1d) which is consistent with our earlier quantification of *Tet* mRNA levels by RT-qPCR in NE cells (Fig. 1g). Comparison of gene expression of *Tet3*^*m/m*^ vs. *Tet3*^*+/+*^ cells identified 2402 DEGs, with nearly equal number of DEGs up- (1306, 54%) and down- (1096, 46%) regulated (Fig. 2b, c). In contrast, comparison of gene expression of *Tet3*^*–/–*^ vs. *Tet3*^*+/+*^ revealed 1071 DEGs where 68% of the DEGs (727) were upregulated and 32% (344) were downregulated, indicating a role for TET3 in gene silencing (Fig. 2b, c). To distinguish the DEGs impacted by the catalytic vs. non-catalytic functions of TET3, we overlapped DEGs in *Tet3*^*m/m*^ and *Tet3*^*–/–*^ cells (Fig. 2d). Genes deregulated in *Tet3*^*m/m*^ cells were influenced by the catalytic activity of Tet3 (i.e., catalytic target genes), while the 749 uniquely deregulated genes in *Tet3*^*–/–*^ cells were genes impacted by non-catalytic roles of TET3 (i.e., non-catalytic target genes) with most of them being upregulated (Fig. 2d). Gene ontology (GO) analysis revealed that the catalytic DEGs were enriched in neural pathways while the non-catalytic DEGs were enriched in mesodermal pathways (Fig. 2e), suggesting that the catalytic and non-catalytic activities of TET3 in NE are involved in controlling the neural and mesoderm programs, respectively. Consistently, key neural transcription factors including *Sox1*, *Neurog2*, and *Hes5* were downregulated in both *Tet3*^*m/m*^ and *Tet3*^*–/–*^ cells while key mesodermal genes such as *Gata6*, *Mcam*, and *Bin1* were aberrantly upregulated in *Tet3*^*–/–*^, but not in *Tet3*^*m/m*^, cells (Fig. 2f and Supplementary Fig. 1e). We also found an increase in expression of cyclin dependent kinase inhibitors *p15*, *p16*, and *p21* only in *Tet3*^*–/–*^ cells (Supplementary Fig. 1f) which is in line with the mild delay in cell cycle progression of these cells (Fig. 1i). Together, these findings support that the catalytic and non-catalytic roles of TET3 activate neural and suppress mesodermal programs, respectively, during specification of NE from ESCs. To confirm that the gene expression changes observed in *Tet3*^*m/m*^ and *Tet3*^*–/–*^ NE cells are due to loss of TET3, we re-expressed *Tet3* in *Tet3*^*m/m*^ and *Tet3*^*–/–*^ cells during differentiation to NE. This ameliorated the expression of both aberrantly upregulated and downregulated genes in these cells (Supplementary Fig. 1g), including key mesodermal genes such as *Hand1* and neural genes such as *Sox5*, supporting that TET3 is responsible for activation and suppression of these genes during NE specification.

### Hyper-DMRs are associated with downregulated neural genes while hypo-DMRs are associated with aberrantly upregulated genes in *Tet3*^*–/–*^ NE cells

**Fig. 3:**
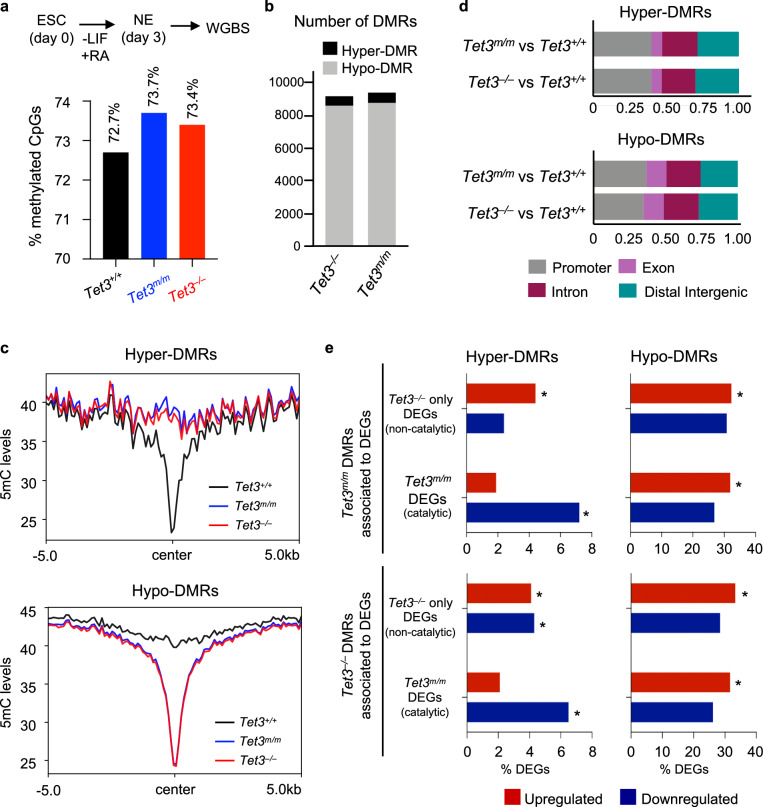
Genome wide analysis of DNA methylation in *Tet3*^*m/m*^, and *Tet3*^*–/–*^ NE cells by WGBS. **a** Percent methylated CpG sites genome-wide in NE cells of indicated genotypes by Whole Genome Bisulfite Sequencing (WGBS). **b** Hyper- and hypo- differentially methylated regions (DMRs = >5 CpGs, methylation difference >20%, and FDR < 0.05) in *Tet3*^*m/m*^ and *Tet3*^*–/–*^ (compared to *Tet3*^*+/+*^) NE cells. **c** DNA methylation levels at all hyper-DMRs (*n* = 924) and all hypo-DMRs (*n* = 12,050) in *Tet3*^*m/m*^ and *Tet3*^*–/–*^ cells. **d** Annotation of hyper- and hypo-DMRs to genomic regions. **e** Percent of catalytic and non-catalytic DEGs that overlap with all hyper- and hypo-DMRs in both *Tet3*^*m/m*^ and *Tet3*^*–/–*^ NE cells. * Hypergeometric test *p*-value < 0.05.

TET3 promotes DNA demethylation, therefore, we analyzed the genome-wide DNA methylation landscape of NE cells (day 3 of –LIF + RA) by whole genome bisulfite sequencing (WGBS) (Supplementary Fig. 2a). As expected, global DNA methylation levels were mildly increased in both *Tet3*^*m/m*^ (73.7%) and *Tet3*^*–/–*^ (73.4%) compared to *Tet3*^*+/+*^ (72.7%) cells at CpG dinucleotides (Fig. 3a). Consistently, we identified ~9000 DMRs in both cell types. However, to our surprise, the vast majority of the DMRs were hypomethylated. Of the 9,222 DMRs in *Tet3*^*m/m*^ cells 626 were hyper- and 8596 were hypo-methylated. Likewise of the 9,335 DMRs in *Tet3*^*–/–*^ cells 571 were hyper- and 8,764 were hypo-methylated (Fig. 3b). Methylation levels were sharply affected at the center of the DMRs (Fig. 3c) and there was a substantial overlap of hyper- or hypo- DMRs between *Tet3*^*m/m*^ and *Tet3*^*–/–*^ cells (Supplementary Fig. 2b, c). The hyper-DMRs were associated to 544 genes in *Tet3*^*m/m*^ and 485 genes in *Tet3*^*–/–*^ cells, with 270 genes common between them (Supplementary Fig. 2b). The hypo-DMRs were associated to 5,989 genes in *Tet3*^*m/m*^ and 5,828 genes in *Tet3*^*–/–*^ cells, with 4283 genes common between them (Supplementary Fig. 2c). Both hyper- and hypo-DMRs were comparably represented throughout the genome at promoters, gene bodies and distal intergenic regions (Fig. 3d). Hyper-DMRs were significantly enriched for downregulated catalytic target genes (*Tet3*^*m/m*^ DEGs) consistent with the role of TET enzymes in DNA demethylation and gene activation (Fig. 3e). Additionally, hypo-DMR associated genes were significantly enriched for upregulated catalytic and non-catalytic (*Tet3*^*–/–*^ only) DEGs (Fig. 3e). These findings suggest that both Tet3 catalytic and non-catalytic mechanisms may regulate DNA methylation dynamics during ESC differentiation.

**Fig. 4:**
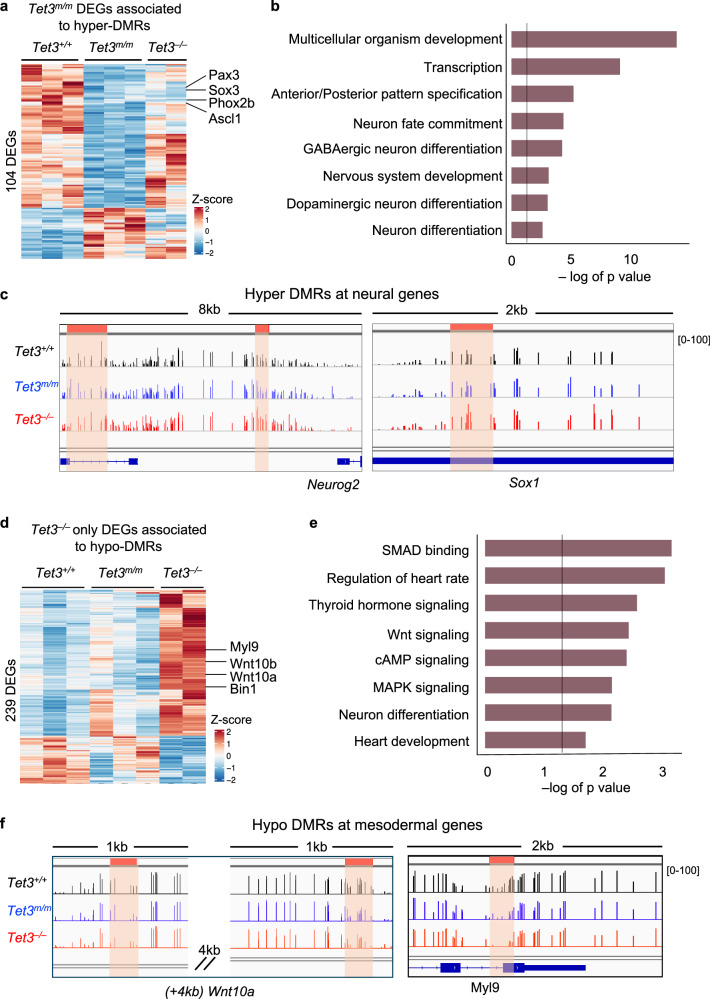
Hyper- and hypo-DMRs are associated to neural and mesodermal genes, respectively. **a** Heatmap of all TET3 catalytic target genes (*n* = 104) associated with hyper-DMRs. **b** GO analysis of hyper-DMR containing TET3 catalytic target genes revealing enrichment for neural terms. Line represents significance (*p* < 0.05). **c** IGV tracks of DNA methylation levels at *Neurog2* and *Sox1* loci (both downregulated in *Tet3*^*m/m*^ and *Tet3*^*–/–*^). Hyper-DMRs are highlighted in orange. **d** Heatmap of all TET3 non-catalytic target genes (*n* = 239) associated with hypo-DMRs. **e** GO analysis of hypo-DMR-containing TET3 non-catalytic target genes revealing enrichment for mesodermal signaling pathways. Line represents significance (*p* < 0.05). **f** IGV tracks of DNA methylation levels at *Wnt10a* and *Myl9* loci (both upregulated in *Tet3*^*–/–*^ but not in *Tet3*^*m/m*^ NEs). Hypo-DMRs are highlighted in orange.

To investigate how DNA hypermethylation impacted gene expression, we overlapped all hyper-DMRs (*n* = 924) with all TET3 catalytic target genes (*Tet3*^*m/m*^ DEGs). We identified 104 hyper-DMR containing catalytic DEGs (Fig. 4a) that were associated with developmental processes including neural differentiation, pattern specification, and transcription (Fig. 4b). This included key neural genes such as *Neurog2* and *Sox1* (Fig. 4c) that are normally upregulated during differentiation of ESCs to NE but were significantly downregulated in both *Tet3*^*m/m*^ and *Tet3*^*–/–*^ cells (Fig. 2f). This is consistent with Tet3 activating neural genes through its catalytic activity of DNA demethylation.

To determine how DNA hypomethylation influenced gene expression, we overlapped all hypo-DMRs identified with TET3 non-catalytic target genes (DEGs unique to *Tet3*^*–/–*^). We identified 239 hypo-DMR containing genes that were mostly upregulated in *Tet3*^*–/–*^ cells (Fig. 4d). They were associated with signaling pathways such as Wnt and mesodermal processes such as heart development (Fig. 4e) and included key mesoderm genes like *Wnt10a* and *Myl9* (Fig. 4f), which were upregulated in *Tet3*^*–/–*^ cells. This suggests that TET3 influences DNA methylation dynamics leading to aberrant upregulation of Wnt signaling and mesodermal programs.

To examine changes in 5hmC levels in *Tet3*^*m/m*^ and *Tet3*^*–/–*^ NE cells, we mapped the genomic distribution of 5hmC by hmeDIP in these cells. Majority of 5hmC was at gene bodies and distal intergenic regions followed by promoters. 5hmC distribution was slightly shifted in *Tet3*^*m/m*^ and *Tet3*^*–/–*^ NE cells with less 5hmC being at promoters (Supplementary Fig. 3a), which is in agreement with the majority of DMRs being at promoters (Fig. 3d). Importantly, consistent with a role for TET3 in DNA hydroxylation the overall 5hmC levels at 5hmC-enriched regions was reduced in *Tet3*^*m/m*^ and *Tet3*^*–/–*^ cells (Supplementary Fig. 3b). This was very notable at gene bodies and distal intergenic regions (Supplementary Fig. 3c, d). We also found that downregulated genes in TET3 deficient NE cells had remarkably reduced levels of 5hmC (Supplementary Fig. 3e). These included several direct targets of TET3 such as *Axin2* and *Pdik1l*, which were not only bound by TET3 in wild type cells but also had reduced levels of 5hmC and were downregulated in *Tet3*^*m/m*^ and *Tet3*^*–/–*^ cells (Supplementary Fig. 3e, f). This data supports that TET3 drives deposition of 5hmC and activation of its direct target genes.

### The maintenance DNA methyltransferase *Dnmt1* is a direct target of TET3 in NE cells

**Fig. 5:**
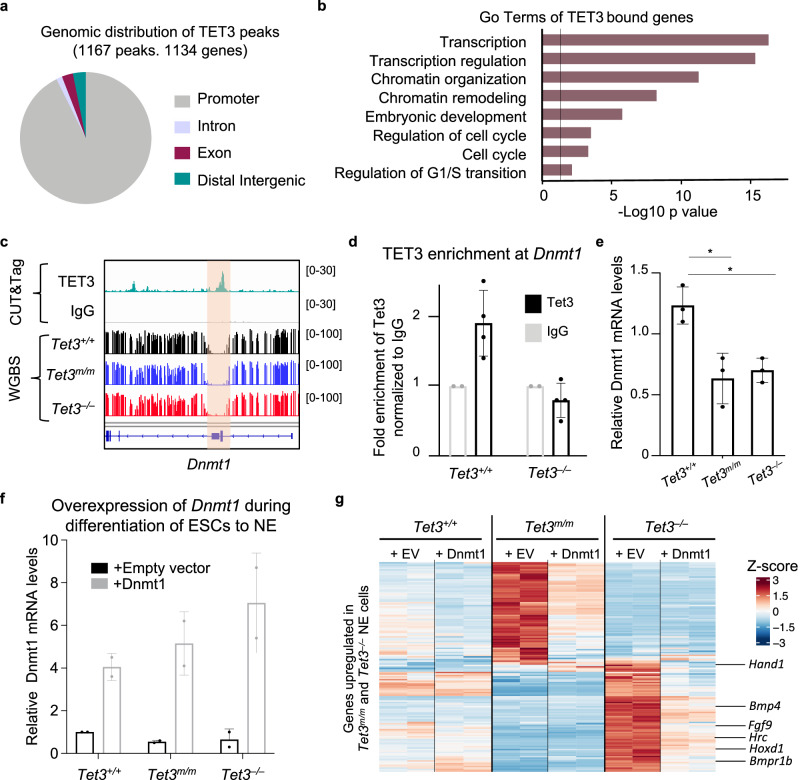
Genome wide occupancy of TET3 in NE cells by CUT&Tag and its enrichment at *Dnmt1* locus. **a** Genomic distribution of 1167 peaks identified by CUT&Tag in *Tet3*^*+/+*^ NE cells. Note, vast majority of the peaks are at promoters. **b** GO analysis of TET3-bound genes. **c** IGV tracks showing TET3 CUT&Tag peaks and DNA methylation levels at *Dnmt1* promoter region. **d** TET3 enrichment at the *Dnmt1* promoter quantified by ChIPqPCR. Data normalized to IgG. **e** Quantification of *Dnmt1* mRNA levels in NE cells (day 3 of –LIF + RA) by RT-qPCR. **f** Quantification of *Dnmt1* by RT-qPCR in NE cells expressing either empty vector (EV) or Dnmt1. **g** Heatmap of upregulated genes in *Tet3*^*m/m*^ and *Tet3*^*–/–*^ NE cells where their expression is corrected by overexpression of *Dnmt1*. For all panels error bars represent SD and * *p* < 0.05 **p < 0.005.

To determine genes directly bound by TET3 in NE cells, we mapped the genomic occupancy of TET3 by CUT&Tag. We identified 1167 peaks genome wide and the majority (92.7%) of them were associated to promoters (+/– 2 kb of TSS; Fig. 5a). The peaks identified annotated to 1,134 genes which were enriched for regulators of transcription, chromatin organization, and cell cycle (Fig. 5b). Because of the low number of peaks identified, we observed only a small overlap of TET3-bound genes to DEGs and hyper- or hypo-DMRs in *Tet3*^*m/m*^ or *Tet3*^*–/–*^ cells (Supplementary Fig. 4a–c). Consistent with a role for TET3 in DNA hydroxylation, we also found that 5hmC levels were reduced at all TET3 bound regions, especially at TET3 bound promoters (Supplementary Fig. 4d, Supplementary Fig. 3f). Among the genes bound by TET3, we found the maintenance methyltransferase *Dnmt1* as a direct target of TET3. Specifically, we found that TET3 binds at the promoter of *Dnmt1* at a region that does not have changes in DNA methylation and hydroxymethylation, suggesting that TET3 might regulate *Dnmt1* independent of DNA methylation (Fig. 5c). We validated TET3 binding at the *Dnmt1* promoter by ChIP-qPCR where TET3 was enriched in *Tet3*^*+/+*^, but not in in the negative control *Tet3*^*–/–*^ cells, confirming the specificity of this enrichment (Fig. 5d). *Dnmt1* was downregulated in *Tet3*^*m/m*^ and *Tet3*^*–/–*^ NE cells (Fig. 5e) while the levels of the de novo methyltransferases *Dnmt3a* and *Dnmt3b* were unaffected (Supplementary Fig. 4e). Reduced *Dnmt1* levels can influence DNA methylation maintenance dynamics during ESC differentiation and may be responsible for the increased occurrence of hypo-DMRs at the aberrantly upregulated genes in *Tet3*^*m/m*^ or *Tet3*^*–/–*^ cells. To examine if Dnmt1 can restore proper expression of upregulated genes, we expressed *Dnmt1* in *Tet3*^*m/m*^ and *Tet3*^*–/–*^ NE cells (Fig. 5f) and analyzed gene expression by RNA-seq. We found that *Dnmt1* overexpression ameliorated the aberrant upregulation of genes in *Tet3*^*m/m*^ and *Tet3*^*–/–*^ NE cells (Fig. 5g). This included expression of several mesodermal genes such as *Hand1* and *Hrc* (Fig. 5g), which were also corrected by re-expression of *Tet3* (Supplementary Fig. 1g). This supports that *Dnmt1*, as a direct target of TET3, contributes to repressing some mesodermal programs during NE specification.

## Discussion

*Tet3*, unlike *Tet1* and *Tet2*, is not expressed in ESCs but is induced upon differentiation. While the catalytic and non-catalytic functions of TET1 and TET2 in ESC biology have been investigated by us and others recently^12,14,35,37^, these dual roles of TET3 in lineage specification have not been well defined. Here, we provide evidence in support of catalytic dependent and independent roles of TET3 in activation of neural and silencing of mesodermal programs, respectively, during differentiation of ESCs to NE. (1) Neural genes were downregulated in both *Tet3*^*m/m*^ and *Tet3*^*–/–*^ NE cells while mesoderm gene were aberrantly upregulated in *Tet3*^*–/–*^ NE cells only. (2) The majority of DMRs in *Tet3*^*m/m*^ and *Tet3*^*–/–*^ NE cells were hypomethylated, which correlated with gene upregulation, while the few hyper-DMRs were linked to downregulated neural genes. (3) *Dnmt1* is directly bound by TET3 in NE cells and is downregulated in *Tet3*^*m/m*^ and *Tet3*^*–/–*^ NE cells, which may be in part responsible for the increased region-specific DNA hypomethylation. Our findings establish that the catalytic dependent and independent roles of TET3 have distinct contributions to NE specification with implications in development.

We identified ~2,400 genes regulated by TET3 catalytic activity and ~750 genes regulated by TET3 non-catalytic roles. In agreement with a prior study, we find that TET3 maintains a balance between neural and mesoderm gene expression programs^11^. Our data further expands upon this study by revealing that the catalytic functions of TET3 activate neural genes while its non-catalytic functions suppress mesodermal genes. Interestingly, it was previously shown that TET3 catalytic activity promotes repression of mesodermal genes via demethylation of the Wnt inhibitor *Sfrp4*, where promoter hypermethylation of *Sfrp4* in TET3 null neural progenitor cells resulted in aberrant activation of Wnt signaling and mesodermal programs^11^. We do not observe hypermethylation nor deregulation of *Sfrp4* in *Tet3*^*m/m*^ and *Tet3*^*–/–*^ NE cells. However, we find robust aberrant upregulation of Wnt pathway and mesoderm genes uniquely in *Tet3*^*–/–*^, but not in *Tet3*^*m/m*^, NE cells supporting that silencing of Wnt signaling during NE specification depends on TET3 non-catalytic functions. Non-catalytic functions of other TETs, in particular TET1, have also been implicated in silencing of Wnt signaling and mesodermal genes suggesting TET catalytic independent roles curtails mesodermal programs as ESCs commit to NE^37^.

While the slight increase in the overall 5mC levels and reduction of 5hmC in *Tet3*^*m/m*^ and *Tet3*^*–/–*^ NE cells is consistent with a role for TET3 in DNA demethylation, the identification of a large number of hypo-DMRs (~8000) in contrast to hyper-DMRs (~1000) in both genotypes was surprising. This has not been observed in TET1 and TET2 deficient ESCs or progenitor cell types^12,14^. The association of hyper-DMRs with neural genes, including *Neurog2* and *Sox1*, supports a catalytic role for TET3 in activating neural programs. The hypo-DMRs were associated to 239 non-catalytic DEGs, of which most were aberrantly upregulated in *Tet3*^*–/–*^, but not in *Tet3*^*m/m*^ cells, and were associated to mesoderm pathways. This suggests that TET3 regulates DNA methylation through catalytic and non-catalytic functions which influences neural and mesodermal programs. Our finding that the maintenance methyltransferase *Dnmt1* is a direct target of TET3 and is downregulated in *Tet3*^*m/m*^ and *Tet3*^*–/–*^ cells may provide a possible explanation to the increased number of hypo-DMRs. The reduced levels of *Dnmt1* alone is sufficient to affect DNA methylation maintenance leading to hypomethylation and gene upregulation irrespective of any mild cell cycle or proliferation defects in Tet3-deficient cells. This is further supported by the fact that overexpression of *Dnmt1* in *Tet3*^*m/m*^ and *Tet3*^*–/–*^ cells corrected the aberrant upregulation of genes. The expression of de novo methyltransferases *Dnmt3a* and *Dnm3b*, were unaffected in *Tet3*^*m/m*^ and *Tet3*^*–/–*^ NE cells suggesting they are unlikely involved. However, we cannot rule out any changes in their subcellular localization and intra nuclear redistribution potentially impacting DNA methylation, a phenomenon that has been reported for DNMT3A in some cell types^40^. Future studies mapping the genome-wide distribution of DNMTs and TETs in NE cells may provide better molecular insights underpinning the hypo-DMRs of TET-deficient cells.

The dual roles of TET3 in balancing neuroectoderm and mesoderm fates may have some developmental ramifications during early lineage specification and embryogenesis. Deficiency of TET3 in mice allows overtly normal early and mid-gestation development, but TET3 null embryos die perinatally for reasons not yet defined^16^. While gross organs, and in particular the nervous system components, are well developed in TET3 null embryos, histopathological and molecular studies are limited^16^. Future generation and comparison of TET3 catalytic deficient and knockout embryos at cellular and molecular levels during early development can provide better in vivo insights into how TET3 regulates neuroectoderm fate and neurodevelopment. Indeed, similar studies focused on TET1 and TET2 comparing knockout vs catalytic mutant mice have revealed their distinct biological contributions to embryogenesis and hematopoiesis^12,35,41^.

This study defines the dual functions of TET3 in NE specification. Our transcriptomic and epigenomic comparisons of TET3 catalytic mutant and TET3 knockout ESCs has provided key insights on how TET3 regulates neural and mesodermal gene programs via its catalytic and non-catalytic functions, respectively. In contrast, the few studies in the literature that have investigated the role of TET3 in the neural lineage^11,25,27^ have only used knockout or knock down approaches which abrogates the entire TET3 protein and fails to dissect its dual catalytic and noncatalytic functions. One of these studies reports a requirement for TET3 in differentiation of neural progenitors to neurons and preventing apoptosis^27^. While this study does not provide mechanistic insights into how TET3 regulates these two processes, it suggests that TET3 does not influence cell death in early neuroectoderm commitment but rather prevents apoptosis of established neural progenitor cells. Consistently, we do not find increased apoptosis or deregulation of apoptotic programs in *Tet3*^*m/m*^ and *Tet3*^*–/–*^ neuroectoderm cells. This is not surprising because neuroectoderm specified from ESCs is a different cell type than establish neural progenitors or terminally differentiated neurons, and therefore TET3 may have different functions in each. Future studies aimed at dissecting the catalytic and noncatalytic roles of TET3 in formation of neurons may elaborate which TET3 functions contribute to neurogenesis. Future studies are also warranted to further understand the mechanisms of how TET3 represses mesodermal genes, perhaps either through directly binding at its target genes and recruiting repressive factors to silence them, as we have reported for TET1 and TET2 in ESCs^12,14^, or indirectly through modulation of signaling pathways^37^. It will also be important to better define the relationship between TETs and DNMTs in shaping the DNA methylation dynamics to influence gene expression. These will be important for understanding epigenetic mechanisms of lineage commitment and development with implications for cancers where TETs and DNMTs are mutated or deregulated.

We note that our TET3 occupancy data is limited to only ~1200 peaks, which is fewer compared to TET1 and TET2 peaks in ESCs and in other cell types^12,14^. The lower number of peaks identified could be due to various biological or technical reasons including: (1) TET3 is expressed at lower levels or is unstable in NE cells, (2) TET3 binds with low affinity to chromatin, and/or (3) TET3 antibodies are not sensitive enough or methods such as CUT&Tag or ChIP-seq are not robust enough for mapping occupancies of large proteins like TET3. All these factors could have contributed to the reduced number of TET3 peaks detected in NE cells, and therefore may have prevented the identification of some key direct target genes of TET3. This may also explain the scarcity of TET3 occupancy data in the literature. Future optimization of antibodies and methodologies may resolve these issues. We also note that TET3 catalytic mutant mRNA levels are significantly reduced in *Tet3*^*m/m*^ NE cells compared to TET3 wildtype mRNA levels in *Tet*^*+/+*^ NE cells. It is possible that the TET3 mutant transcript is unstable and that TET3 mutant protein is reduced as well. Using the same conserved mutations in the catalytic pocket of TET1 and TET2 has not resulted in any significant reduction of mutant TET1 or TET2 transcripts^12,14^. Thus, this reduction in TET3 mutant transcript levels is unique to TET3. An alternative possibility that can explain the reduced *Tet3* transcript levels in *Tet3*^*m/m*^ NE cells is an auto-regulatory mechanism, where TET3 catalytic activity is needed for proper expression of *Tet3*. While our CUT&Tag data does not show enrichment of TET3 at *Tet3* promoter and gene body, it is possible that TET3 transiently binds to these regions, or that TET3 is enriched at regions distal to the *Tet3* gene that are not yet implicated in *Tet3* regulation. Regardless, the reduced Tet3 transcript levels in *Tet3*^*m/m*^ NE cells should be taken into consideration in data interpretation.

## Materials and Methods

### Generation of *Tet3*^*m/m*^ and *Tet3*^*–/–*^ mouse ESCs

TET3 catalytic deficient (*Tet3*^*m/m*^) and knockout (*Tet3*^*–/–*^) mouse ESCs were generated following our previous CRISPR/Cas9 gene targeting protocols^42^. To generate *Tet3*^*m/m*^ mouse ESCs: A donor oligo containing two point mutations (H950Y and D952A) in the TET3 iron binding site (in exon 9) and a silent mutation introducing a *HaeIII* restriction enzyme site was synthesized. A gRNA targeting exon 9 was designed and cloned into a px330-GFP vector. 1.5 μg of gRNA and 3.5 μg of donor oligo were transfected into wild-type mouse ESCs (v6.5 mixed 129/B6 background, male). Correctly targeted clones were validated by RLFP using *HaeIII* restriction enzyme and sanger sequencing. To generate *Tet3*^*–/–*^ mouse ESCs: two gRNA flanking exon 4 were generated and cloned into px330-GFP and -Cherry vectors. 2.5 μg of each gRNA was transfected into wild-type ESCs, and double positive cells were sorted by FACs. Correctly targeted clones were validated by PCR and loss of *Tet3* mRNA expression was confirmed by RT-qPCR. Sequences of gRNA, oligos, and primers used for genotyping are in Supplementary Table 1. The *Tet3*^*m/m*^ and *Tet3*^*–/–*^ mouse ESCs generated in this study are available upon request from the corresponding author.

### Mouse ESC culture and differentiation to NE

All mouse ESCs (v6.5 mixed129/B6 background, male) were cultured on feeders in ESC media containing serum/LIF as previously described^12^. For gene targeting validation, ESCs were trypsinized, pre-plated for 1 h to remove feeder cells, and seeded on gelatin-coated plates for 24 h before harvesting for DNA and RNA extraction. ESCs were differentiated to NE as previously described^39^. Briefly, pre-plated ESCs were cultured on gelatin-coated plates for 24 h and differentiated for 72 h in the absence of LIF (DMEM supplemented with 10% FBS, 2 mM glutamine, 1x non-essential amino acids, 100 U/mL penicillin, 100 g/mL streptomycin, 1 μM retinoic acid (RA), and 50 mM β-mercaptoethanol). Cells were harvested at 72 h of differentiation for transcriptomic and epigenomic analyzes.

### Transfection of mouse ESCs during differentiation

Hemagglutinin (HA)–tagged wild-type Tet3 catalytic domain (Tet3-CD) was cloned into FUW-HA-2A-tdTomato vector. Myc-tagged Dnmt1 (pCAG-myc-Dnmt1-IRES-blast) and empty vector (pCAG-myc-IRES-blast) were obtained from Dr. Taiping Chen at University of Texas MD Anderson Cancer Center. ESCs were cultured and differentiated as stated above. All transfections were carried out using XtremeGene DNA transfection reagent following manufacturer guidelines. For Tet3-CD overexpression: *Tet3*^*+/+*^, *Tet3*^*m/m*^, and *Tet3*^*–/–*^ cells were transfected with either empty vector (FUW-HA-2A-tdTomato) or Tet3-CD (FUW-HA-Tet3-CD-2A-tdTomato) on day 2 of -LIF + RA differentiation. Cells were collected for RNA extraction 48 h later when Tet3-CD expression and tdTomato+ cells were the highest. For *Dnmt1* overexpression: *Tet3*^*+/+*^, *Tet3*^*m/m*^, and *Tet3*^*–/–*^ ESCs were transfected with either empty vector or myc-Dnmt1 and media was changed to -LIF + RA at day 0. 48 h after transfection, cells were treated with 10 ug blasticidin for 24 h followed by collection for RNA extraction. RNA was extracted using Qiagen RNeasy kit and subjected to RNA-seq at Novogene.

### Proliferation and cell cycle assay

For the proliferation assays, 25,000 ESCs of each genotype were seeded onto one well of a gelatin-coated 12-well plate. After 24 h, media was changed to differentiation media (–LIF + RA) and viable cells were counted every 24 h for 3 days. For cell cycle analysis, day 3 differentiated cells (–LIF + RA) were treated with 20uM EdU and 0.1 mg/mL RNase A and 50 µg/mL propidium iodide (PI, P4864, SigmaAldrich, Inc., St. Louis, MO, USA) using the Click-It 647 EdU kit (Thermo-Fisher C10424) following manufacturer’s instructions. Cell cycle analysis was performed using a BD LSR II flow cytometer (BD Biosciences, Franklin Lakes, NJ, USA) and FlowJo software (v.10.8.0).

### Teratoma Assay

Pre-plated wild-type, *Tet3*^*m/m*^, and *Tet3*^*–/–*^ mouse ESCs were grown on gelatin-coated plates for 24 h. 1 million cells were harvested and injected subcutaneously into SCID mice (Taconic) as previously described^12^. After 7 weeks, tumors were harvested, fixed in formalin for 2 days, paraffin embedded, sectioned, and stained with Hematoxylin and Eosin at the Einstein Histopathology Core. Slide were imaged using an upright light microscope.

### RT-qPCR and ChIP-qPCR

RT-qPCR was performed as previously described^12^. RNA was extracted from 3 clones of each genotype at day 0 (ESCs cultured on gelatin-coated plates for 24 h) and day 3 of –LIF + RA differentiation using the Qiagen RNeasy mini kit (74104) and subjected to cDNA synthesis using Superscript IV kit (Invitrogen, 18091050), according to manufacturer’s protocols. RT-qPCR was performed using SYBR green and primers in Supplementary Table 2 in a BD Applied Biosystems StepOne^TM^ Real-Time PCR System. Data were normalized to *Gapdh* and wild-type controls. ChIP-qPCR for TET3 was performed in day 3 -LIF + RA NE cells using anti-TET3 antibody (Millipore ABE290) and primers targeting *Dnmt1* promoter as described before^12^. Dnmt1 primers were designed according to TET3 bound coordinates identified from CUT&Tag analysis. Data was calculated as fold enrichment over IgG and plotted as fold change.

### RNA-Seq and data analysis

RNA-seq was performed as previously described^12^. Day 3 -LIF + RA neuroectoderm cells were collected and total RNA was extracted as described above. Library preparation and mRNA sequencing were done by Novogene using their Illumina Novoseq 6000 platform. Trim galore (v0.6.7) was used to remove adapters and trimmed reads were mapped to the mouse genome (mm39) using STAR (v2.7.9a)^43^ with default parameters. Gene counts were extracted from mapped reads using featureCounts with --largestOverlap parameter. Raw counts were used to identify differentially expressed genes (DEGs) with DESeq2^44^ (false discovery rate-adjusted p-value < 0.05 and fold-change >1.5), following the package documentation. Gene ontology (GO) enrichment analyzes of DEGs was performed using DAVID^45^ (https://david.ncifcrf.gov/). All plots were made in R using custom scripts.

### WGBS and data analysis

Whole-genome bisulfite sequencing (WGBS) was performed as described previously^12^. DNA from day 3 -LIF + RA NE cells was extracted by Quick-DNA miniprep kit (Zymo, D3024) following the manufacturer’s instructions. Bisulfite conversion and sequencing were performed at BGI Genomics (https://en.genomics.cn/). Lamda DNA spike-in confirmed a >99.0% bisulfite conversion efficiency. The libraries were subjected to 100 bp pair-end sequencing on a HiSeq 4000 Illumina platform. Raw reads were filtered by SOAPnuke (https://github.com/BGI-flexlab/SOAPnuke) with the parameters -n 0.001 -l 20 -q 0.4 –adaMR 0.25 –ada_trim –polyX 50 –minReadLen 100 to remove adapters and filter out low-quality reads. Clean reads were mapped to mouse genome mm10 using Bismark (v0.23.1)^46^ with default parameters. Duplicated reads were removed using deduplicate_bismark and methylation status of each cytosine was determined with bismark_methylation_extractor. The deduplicated BAM files were balanced to the sample with the lowest reads using samtools. The resulting BAM files were then converted to be compatible with methpipe^47^ (v5.0.1) using methpipe format_reads -f and sorted using samtools. Bisulfite rate conversion was determined by bsrate, single-site methylation levels were identified by methcounts, and symmetric CpGs were merged with symmetric-cpgs. Methylation was compared between samples using methdiff and differentially methylated regions (DMRs, regions >5 CpGs, methylation difference >20%, and FDR < 0.05) between *Tet3*^*m/m*^ vs. *Tet3*^*+/+*^ and *Tet3*^–/–^ vs. *Tet3*^*+/+*^ were identified using hmr and then dmr. DMRs were annotated to genomic features with R package ChIPseeker (v1.36.0). Methylation line plots at DMRs were generated using plotProfile function of deepTools (v3.5.1)^48^. For visualization on Integrative Genome Browser (IGV), bedGraph files were converted to bigwig files using bedGraphToBigWig from bedtools (v2.30.0)^49^. To determine the overlapping *Tet3*^*m/m*^ and *Tet3*^*–/–*^ DMRs, BED files were intersected using bedtools intersect with default parameters.

### hmeDIP and data analysis

*Tet3*^*+/+*^, *Tet3*^*m/m*^ and *Tet3*^*–/–*^ mouse ESCs were differentiated to NE (-LIF + RA for 3 days). DNA was extracted and subjected to hMeDIP (Active Motif hMeDIP Kit #55010) following manufacturer guidelines, and amplified using NEBNext HiFi 2x PCR Master mix. Libraries were cleaned with AMPure XP beads (#A63880) and subjected to 75 bp paired-end sequencing using Illumina NextSeq 500 platform at the Einstein Epigenomics Core. Adapters were trimmed using TrimGalore and sequencing reads were mapped to the mouse genome (mm10) using bowtie2 (v2.4.5)^50^ with the following parameters: --local --sensitive --very-sensitive-local --no-unal --no-mixed --no-discordant –phred33 -I 10 -X 700. The BAM files were balanced to the sample with the lowest reads using samtools and were used to call peaks using MACS2 (v2.2.7.1)^51^ with the following parameters: -p 0.0001 -f BAMPE --keep-dup all. BED files with genomic coordinates were annotated using ChIPseeker (v1.36.0). IgG bigwig was subtracted from *Tet3*^*+/+*^, *Tet3*^*m/m*^ and *Tet3*^*–/–*^ bigwigs using function bigwigCompare --operation subtract from deepTools. The output bigwig files were used to make line plots using function plotProfile from deepTools.

### CUT&Tag and data analysis

To map the genomic occupancy of TET3, CUT&Tag was applied to day 3 -LIF + RA differentiated wild-type cells as described previously^12^. 500,000 cells (per antibody) were collected at 72 h of differentiation and crosslinked with 0.5% formaldehyde for 5 mins. Fixed cells were bound to Concavalin A-coated beads, permeabilized, and incubated with primary antibodies (anti-Tet3 Millipore ABE290 and rabbit IgG isotype control CST 3900) overnight at 4 °C. Samples were then incubated with secondary antibody (guinea pig anti-rabbit Antibodies Online ABIN101961) at room temperature followed by incubation with pre-loaded pA-Tn5. Transposase was activated at 37 °C with magnesium to fragment bound DNA. DNA was isolated by phenol-chloroform isoamyl alcohol extraction and amplified using NEBNext HiFi 2x PCR Master mix. Libraries were cleaned with AMPure XP beads (#A63880) and subjected to 75 bp paired-end sequencing using Illumina NextSeq 500 platform at the Einstein Epigenomics Core. Sequencing reads were mapped to the mouse genome (mm10) using bowtie2 (v2.4.5) with the following parameters: --local --sensitive --very-sensitive-local --no-unal --no-mixed --no-discordant –phred33 -I 10 -X 700. Duplicates were removed using Picard tools MarkDuplicates function. The BAM files were balanced to the sample with the lowest reads using samtools and were used to call peaks using MACS2 (v2.2.7.1) with the following parameters: -p 0.00001 -f BAMPE --keep-dup all. BED files with genomic coordinates were annotated using ChIPseeker (v1.36.0). Line plots and heatmaps were generated using deepTools.

### Statistics and reproducibility

All experiments were performed with three biological replicates unless otherwise described in the methods. GraphPad Prism 8 was used to perform statistical analyzes. One way ANOVA test or unpaired t-test was applied to calculate statistical significance. Differences were considered statistically significant when *p* < 0.05. Statistical methods used for bioinformatics analyzes are explained in detail under the respective methods sub-sections.

### Reporting summary

Further information on research design is available in the Nature Portfolio Reporting Summary linked to this article.

### Supplementary information

Supplemental Material

Description of Additional Supplementary Files

Supplementary Data 1

Reporting Summary

## Data Availability

The RNA-seq, CUT&Tag and WGBS data have been deposited in the Gene Expression Omnibus (GEO) database. Accession number (GSE237800). The uncropped images of gels can be found under Supplementary Information as Supplementary Fig. 5. The source data for all figures are provided in Supplementary Data 1. All other relevant data and the ESC lines used to generate the data in this study are available upon request from the corresponding author.
