# Supplementary material for: Catalytic-dependent and -independent roles of TET3 in the regulation of specific genetic programs during neuroectoderm specification
Source: Commun Biol. 2024 Apr 5;7:415. doi: 10.1038/s42003-024-06120-w (PMC10997653; doi:10.1038/s42003-024-06120-w)
Supplement: Supplementary file 1 — Supplemental Material [file 42003_2024_6120_MOESM1_ESM.pdf]

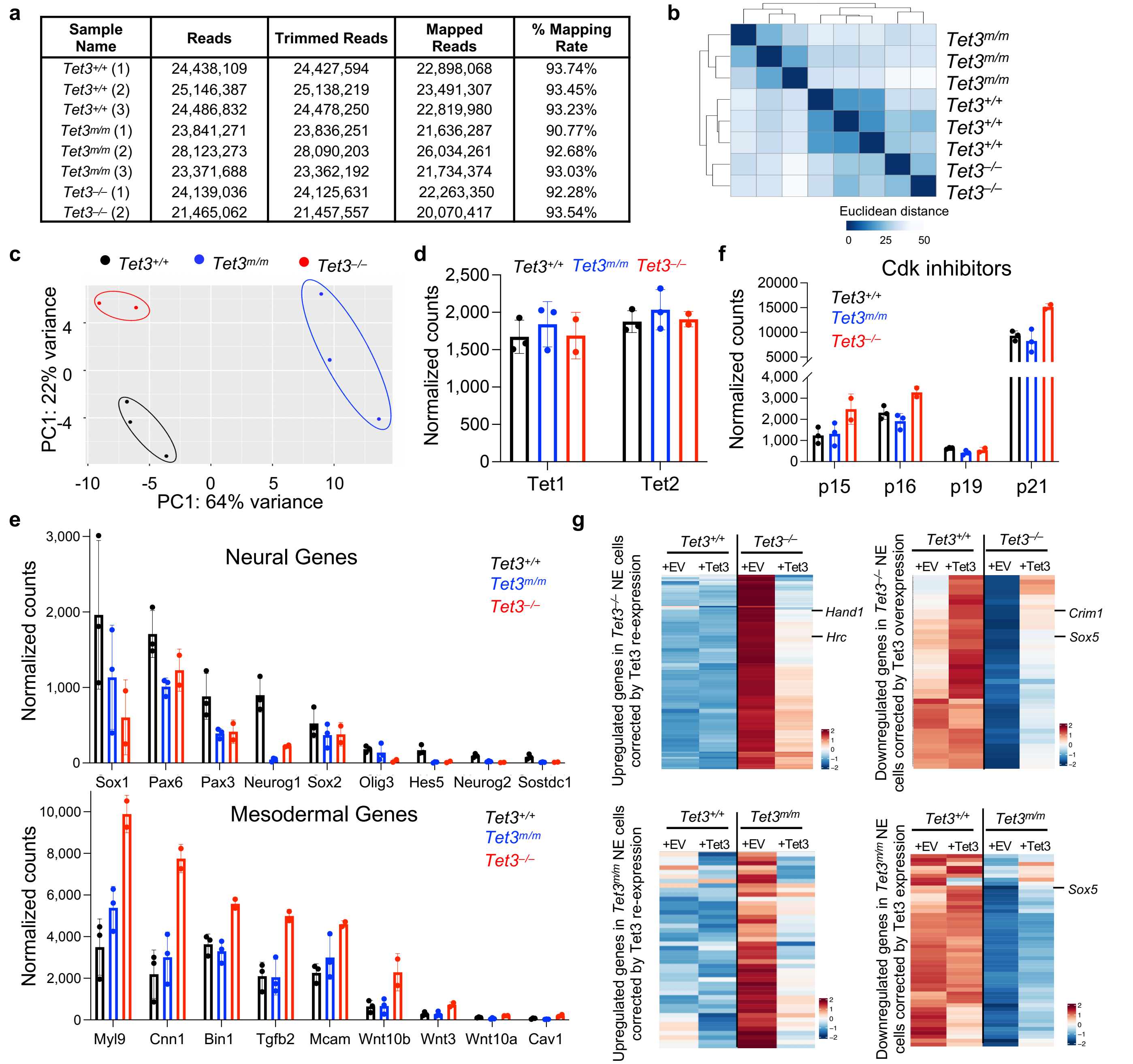

Sample Name	Reads	Trimmed Reads	Mapped Reads	% Mapping Rate
$Tet3^{+/+}$ (1)	24,438,109	24,427,594	22,898,068	93.74%
$Tet3^{+/+}$ (2)	25,146,387	25,138,219	23,491,307	93.45%
$Tet3^{+/+}$ (3)	24,486,832	24,478,250	22,819,980	93.23%
$Tet3^{m/m}$ (1)	23,841,271	23,836,251	21,636,287	90.77%
$Tet3^{m/m}$ (2)	28,123,273	28,090,203	26,034,261	92.68%
$Tet3^{m/m}$ (3)	23,371,688	23,362,192	21,734,374	93.03%
$Tet3^{-/-}$ (1)	24,139,036	24,125,631	22,263,350	92.28%
$Tet3^{-/-}$ (2)	21,465,062	21,457,557	20,070,417	93.54%

Supplementary Figure 1: RNA-seq analysis of *$Tet3^{m/m}$* and *$Tet3^{-/-}$* neuroectoderm cells

a. Summary of total, trimmed and mapped reads, and mapping rate per sample.

b. Sample distribution plot showing that samples of the same genotype cluster together.

c. Principle component analysis (PCA) of RNA-seq data showing that samples of the same genotype cluster together.

d. RNA-seq normalized counts of *Tet1* and *Tet2*. Note, neither Tet1 nor Tet2 are deregulated. Error bars represent standard deviation.

e. Plot of RNA-seq normalized counts of selected neural (top) and mesodermal (bottom) genes. Neural genes are downregulated in both *$Tet3^{m/m}$* and *$Tet3^{-/-}$* cell lines while mesodermal genes are uniquely upregulated in *$Tet3^{-/-}$* cells. Error bars represent standard deviation.

f. Plot of RNA-seq normalized counts of cyclin dependent kinase inhibitor (Cdkn) genes. Note *p15*, *p16*, and *p21* are uniquely upregulated in *$Tet3^{-/-}$* cells. Error bars represent standard deviation.

g. Heatmap of up and downregulated genes in *$Tet3^{m/m}$* and *$Tet3^{-/-}$* cells that are ameliorated by re-expression of TET3 catalytic domain. Empty vector (EV) is used as control.

a

Sample Name	Reads	Trimmed Reads	Mapped Reads	% Mapping Rate	After Deduplicate	% Duplicate
Tet3$^{+/+}$	514,498,240	514,497,215	378646820	73.60%	341,578,304	9.79%
Tet3$^{m/m}$	540,538,184	540,537,178	394296833	72.95%	355,789,910	9.77%
Tet3$^{–/–}$	473,848,041	473,847,181	334638862	70.62%	303,987,670	9.16%

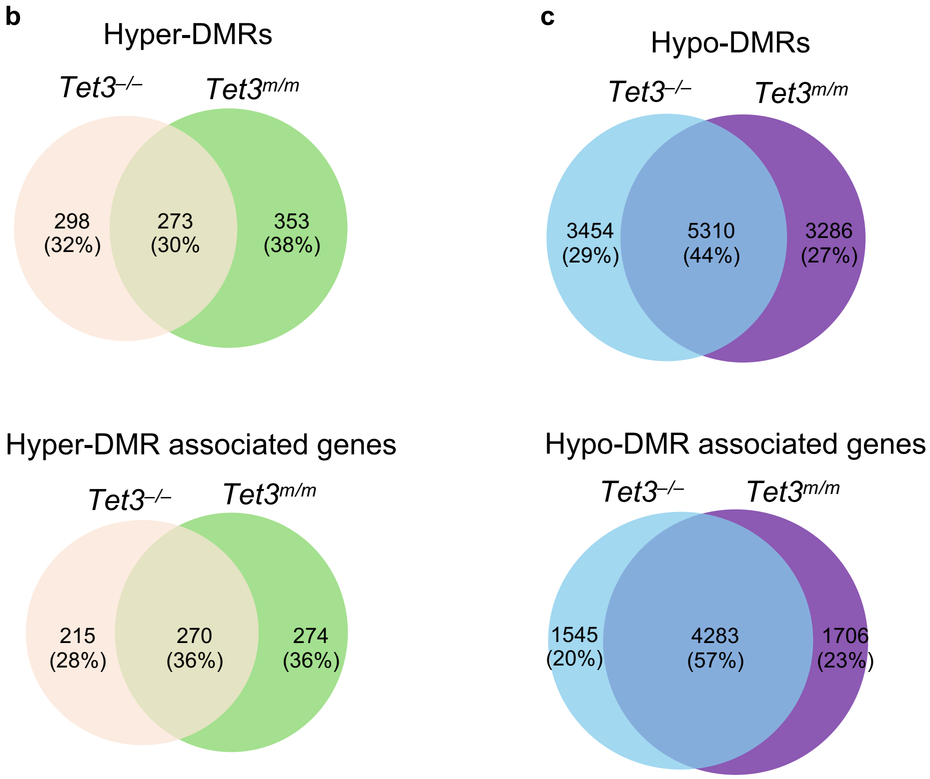

Supplementary Figure 2: Genome wide mapping of DNA methylation in *Tet3*$^{m/m}$ and *Tet3*$^{–/–}$ neuroectoderm cells by WGBS.

a. Summary of total, trimmed and mapped reads, mapping rate, and duplication per sample.

b. Overlap of hyper-DMRs (top) and hyper-DMR-associated genes (bottom) identified using methpipe.

c. Overlap of hypo-DMRs(top) and hypo-DMR-associated genes (bottom).

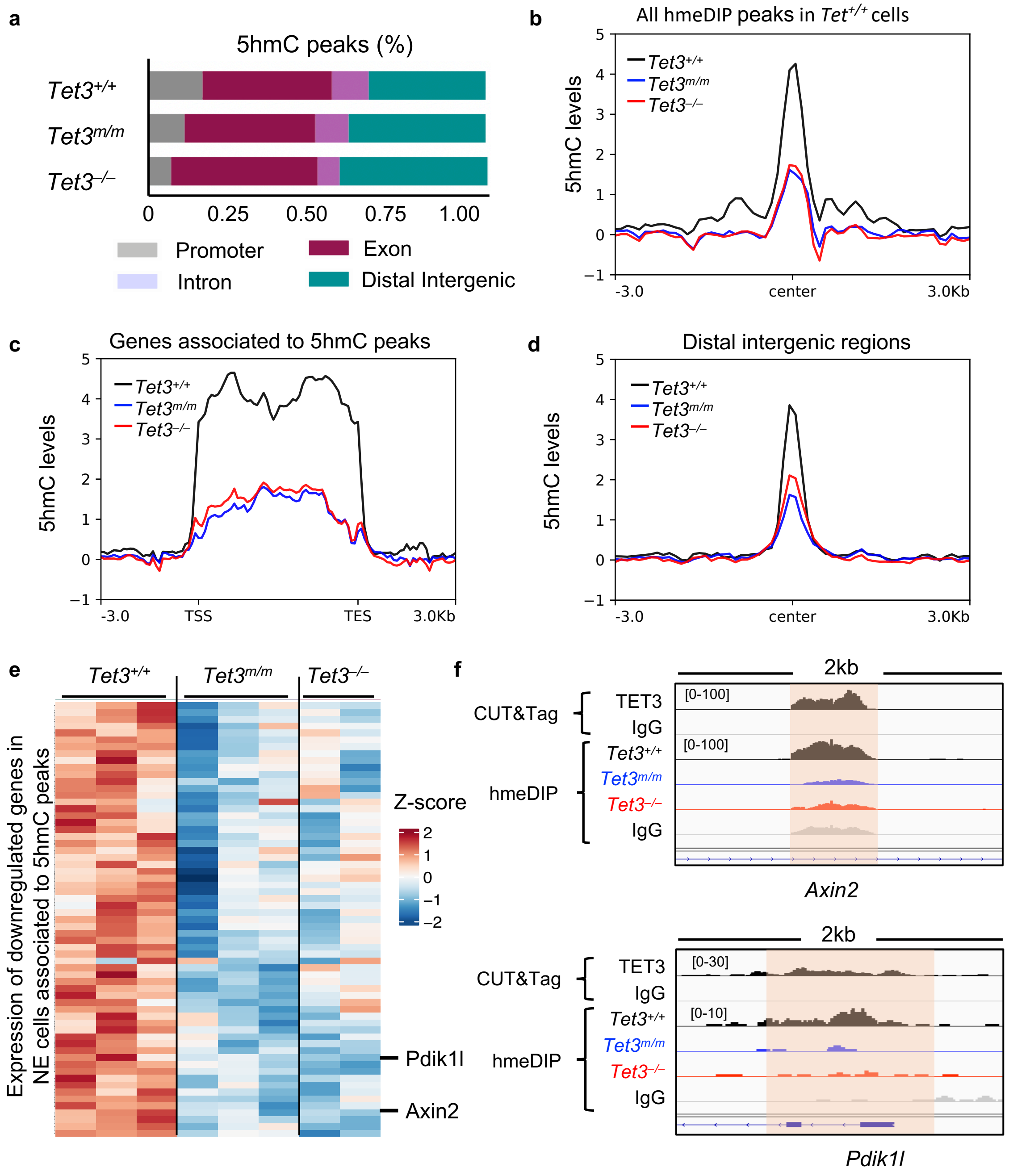

Supplementary Figure 3: Analysis 5hmC by hmeDIP in *$Tet3^{+/+}$*, *$Tet3^{m/m}$ and $Tet3^{-/-}$* NE cells

a. Genomic distribution of 5hmC peaks in *$Tet3^{+/+}$*, *$Tet3^{m/m}$* and *$Tet3^{-/-}$* cells.

b. 5hmC levels at all hmeDIP peaks in *$Tet3^{+/+}$* cells. Note that 5hmC levels at these peaks are reduced in *$Tet3^{m/m}$* and *$Tet3^{-/-}$* cells.

c. 5hmC levels across transcription start site (TSS) to transcription end site (TES) of genes.

d. 5hmC levels across distal intergenic regions.

e. Heatmap of genes associated to 5hmC peaks in wild type NE cell. Note that these genes are downregulated in *$Tet3^{m/m}$* and *$Tet3^{-/-}$* cells.

f. Genome browser tracks showing 5hmC levels at TET3-enriched regions at representative loci.

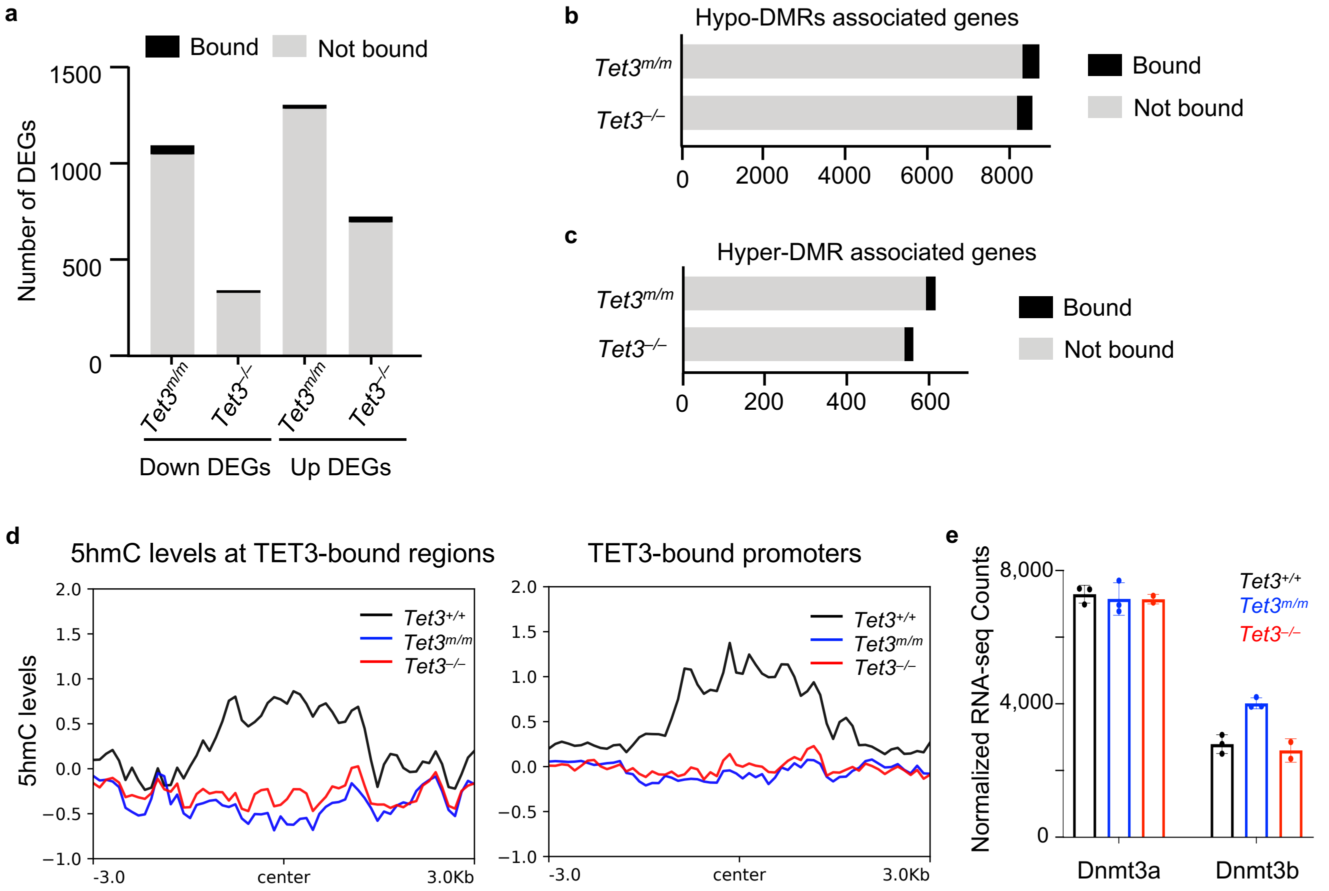

Supplementary Figure 4: Summary of TET3 CUT&Tag in *Tet3*$^{+/+}$ neuroectoderm cells

a. Number of DEGs bound by TET3.

b. Number of hypo-DMR associated genes bound by TET3.

c. Number of hyper-DMR associated genes bound by TET3.

d. 5hmC levels at all TET3 bound regions (left) and at TET3 bound promoters (right).

e. RNA-seq normalized counts of *Dnmt3a* and *Dnmt3b* in NE cells. Count numbers are not significant between genotypes based on DESeq2. Error bars represent standard deviation.

a

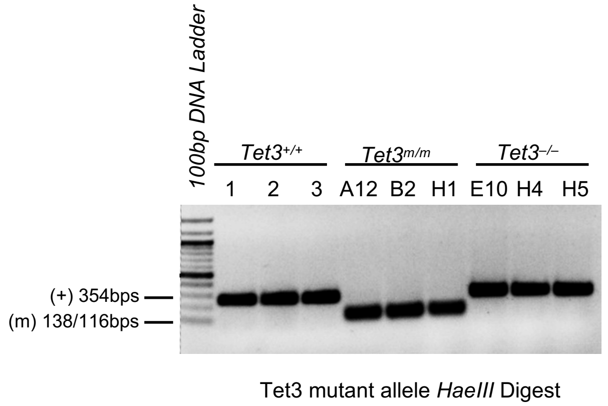

b

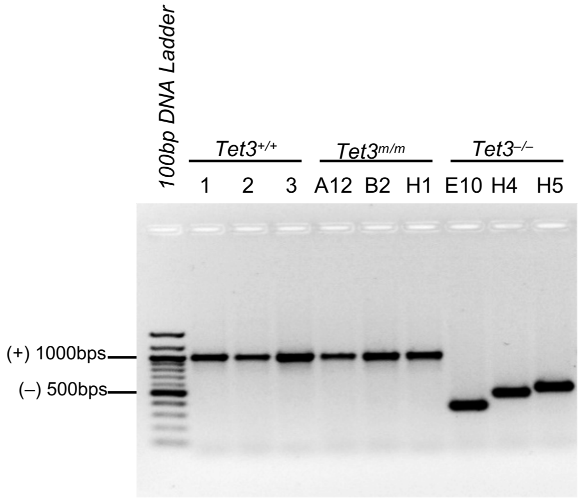

Supplementary Figure 5: Uncropped images of gels shown in Fig. 1b

a. Genotyping of Tet3 mutant cells by RFLP with *HaeIII.*

b. Genotyping of Tet3 knockout cells by PCR with primers flanking exon 4.

Supplementary Table 1: List of oligonucleotides used in this study

Name	Sequence	Purpose	Source
Tet3 Mut exon 9 gRNA For	CCTAAATGTTCTCATCCTGG	Mutating Tet3 exon 9	This paper
Tet3 Mut exon 9 gRNA Rev	CCAGGATGAGAACATTTAGG	Mutating Tet3 exon 9	This paper
Tet3 Mut gene block sequence	5'agctatcgGAATTCTGT…500bp….CATGGACTTCTGT GCCCACGCC**TACAAGGCC**CAACATAACCTCTACAA TGGGTGCACTGTGGTA…410bp…..Ctcgatcgt3'	Introducing HKD to YKA mutation the catalytic pocket of Tet3 in exon 9	This paper
Tet3 Mut Genotyping For	ATTCACACAGGTGACCAATGAG	Genotyping Tet3 Mut allele	This paper
Tet3 Mut Genotyping Rev	CCTAGGTAGTGTTGGCTTAGAT	Genotyping Tet3 Mut allele	This paper
Tet3 KO exon 4 left gRNA For	TCCTAAATGTTCTCATCTGG	Deletion of Tet3 Exon 4	This paper
Tet3 KO exon 4 left gRNA Rev	CCAGATGAGAACATTTAGGA	Deletion of Tet3 Exon 4	This paper
Tet3 KO exon 4 right gRNA For	TTCCCACAGCCTGGTTTTCT	Deletion of Tet3 Exon 4	This paper
Tet3 KO exon 4 right gRNA Rev	AGAAAACCAGGCTGTGGGAA	Deletion of Tet3 Exon 4	This paper
Tet3 KO Genotyping For	CTTAAGCAAAGCTCCCATGC	Genotyping Tet3 KO allele	This paper
Tet3 KO Genotyping Rev	GACTCTGGGTATTTGGGGGT	Genotyping Tet3 KO allele	This paper
Tet3 exon 3 RTqPCR For	CCGTGACTGTGCTCTCAACT	Real time qPCR	Reference #15
Tet3 exon 4 RTqPCR Rev	CCATGAGTTCCCGGATAGAA	Real time qPCR	Reference #15
Tet2 exon 3 RTqPCR For	CAAGCAACCAAAAGCACAGA	Real time qPCR	Reference #14
Tet2 exon 4 RTqPCR Rev	GGACCAGCTCCTAGATGGGTA	Real time qPCR	Reference #14
Tet1 RTqPCR For	TGCACCTACTGCAAGAATCG	Real time qPCR	Reference #13
Tet1 RTqPCR Rev	AAATTGGCATCACAGCTTCC	Real time qPCR	Reference #13
Dnmt1 RTqPCR For	GCTACCAGTGCACCTTTGGT	Real time qPCR	Reference #46
Dnmt1 RTqPCR Rev	CAGAGGCAGCTTTTCTCCTG	Real time qPCR	Reference #46
Gapdh RTqPCR For	ACATCTCACTCAAGATTGTCAGCA	Real time qPCR	Reference #10
Gapdh RTqPCR Rev	ATGGCATGGACTGTGGTCAT	Real time qPCR	Reference #10

Supplementary Table 2: List of reagents and resources used in this study

Reagent/Resource	Source	Identifier
**Antibodies**		
Anti-Tet3	Millipore	ABE290
Rabbit IgG isotype control DA1E	CST	3900
Guinea pig anti-rabbit	Antibodies Online	ABIN101961
**Kits**		
Qiagen RNeasy Mini kit	Qiagen	74104
Quick-DNA miniprep kit	Zymo	D3024
Superscript IV first strand	Invitrogen	18091050
XtremeGene 9 DNA transfection reagent	Roche	06365787001
Click-It 647 EdU	Thermo Fisher	C10424
MinElute PCR Purification Kit	Qiagen	28004
hmeDIP kit	Active Motif	55010
**Primers/Oligos**		
RT-qPCR primers	Table S1	N.A.
Genotyping Primers	Table S1	N.A.
gRNAs for gene targeting	Table S1	N.A.
**Plasmids**		
Topo pcr2.1 Tet3 mutant donor vector	This paper	N.A.
pX330-Tet3-gRNA	This paper	N.A.
pCAG-Myc-IRESblast	From Taiping Chen Lab	N.A
pCAG-Myc-Dnmt1-IRESblast	From Taiping Chen Lab	N.A
FUW-HA-2A-tdTomato	Reference #46	N.A
FUW-HA-Tet3CD-2A-tdTomato	This Paper	N.A
**Software**		
Trim galore v0.6.7	Babraham Institute	https://www.bioinformatics.babraham.ac.uk/projects/trim_galore/
STAR v2.7.9a	Reference #48	https://github.com/alexdobin/STAR
DESeq2 v1.40.2	Reference #49	http://www.bioconductor.org/packages/release/bioc/html/DESeq2.html
DAVID 6.8	Reference #50	https://david.ncifcrf.gov
Bismark v0.23.1	Reference #51	https://github.com/FelixKrueger/Bismark
Methpipe v5.0.1	Reference #52	http://smithlabresearch.org/software/methpipe/
ChIPseeker v1.36.0	Github	https://guangchuangyu.github.io/software/ChIPseeker/
Bedtools v2.30.0	Reference #54	https://bedtools.readthedocs.io/en/latest/
deepTools v3.5.1	Reference #53	https://deeptools.readthedocs.io/en/develop/index.html
Bowtie2 v2.4.5	Reference #55	http://bowtie-bio.sourceforge.net/bowtie2/index.shtml
Integrative Genomics Viewer v2.14.1	Broad Institute	http://software.broadinstitute.org/software/igv/
MACS v2.2.7.1	Reference #56	https://github.com/macs3-project/MACS
GraphPad Prism 8 v8.4.2	GraphPad	https://www.graphpad.com/
