# Supplementary material for: Catalytic-dependent and -independent roles of TET3 in the regulation of specific genetic programs during neuroectoderm specification
Source: Commun Biol. 2024 Apr 5;7:415. doi: 10.1038/s42003-024-06120-w (PMC10997653; doi:10.1038/s42003-024-06120-w)
Supplement: Supplementary file 2 — Description of Additional Supplementary Files [file 42003_2024_6120_MOESM2_ESM.pdf]

Description of Additional Supplementary Files

File name: Supplementary Data 1

Description: The source data behind the graphs and heatmaps in the paper.
