# Supplementary material for: Catalytic-dependent and -independent roles of TET3 in the regulation of specific genetic programs during neuroectoderm specification
Source: Commun Biol. 2024 Apr 5;7:415. doi: 10.1038/s42003-024-06120-w (PMC10997653; doi:10.1038/s42003-024-06120-w)
Supplement: Supplementary file 4 — Reporting Summary [file 42003_2024_6120_MOESM4_ESM.pdf]

nature portfolio

Corresponding author(s): Meelad Dawlaty

Last updated by author(s): Mar 20, 2024

Reporting Summary

Nature Portfolio wishes to improve the reproducibility of the work that we publish. This form provides structure for consistency and transparency in reporting. For further information on Nature Portfolio policies, see our Editorial Policies and the Editorial Policy Checklist.

Statistics

For all statistical analyses, confirm that the following items are present in the figure legend, table legend, main text, or Methods section.

n/a	Confirmed	
☐	☒	The exact sample size (*n*) for each experimental group/condition, given as a discrete number and unit of measurement
☐	☒	A statement on whether measurements were taken from distinct samples or whether the same sample was measured repeatedly
☐	☒	The statistical test(s) used AND whether they are one- or two-sided<br>*Only common tests should be described solely by name; describe more complex techniques in the Methods section.*
☒	☐	A description of all covariates tested
☒	☐	A description of any assumptions or corrections, such as tests of normality and adjustment for multiple comparisons
☐	☒	A full description of the statistical parameters including central tendency (e.g. means) or other basic estimates (e.g. regression coefficient) AND variation (e.g. standard deviation) or associated estimates of uncertainty (e.g. confidence intervals)
☐	☒	For null hypothesis testing, the test statistic (e.g. *F*, *t*, *r*) with confidence intervals, effect sizes, degrees of freedom and *P* value noted<br>*Give P values as exact values whenever suitable.*
☒	☐	For Bayesian analysis, information on the choice of priors and Markov chain Monte Carlo settings
☒	☐	For hierarchical and complex designs, identification of the appropriate level for tests and full reporting of outcomes
☐	☒	Estimates of effect sizes (e.g. Cohen's *d*, Pearson's *r*), indicating how they were calculated

Our web collection on statistics for biologists contains articles on many of the points above.

Software and code

Policy information about availability of computer code

Data collection	All resources, software and codes used in this study for data collection are commercially and publicly available as open source and are detailed in the methods and listed in supplementary table 2.
Data analysis	All resources, software and codes used in this study for data collection are commercially and publicly available as open source and are detailed in the methods and listed in supplementary table 2.

For manuscripts utilizing custom algorithms or software that are central to the research but not yet described in published literature, software must be made available to editors and reviewers. We strongly encourage code deposition in a community repository (e.g. GitHub). See the Nature Portfolio guidelines for submitting code & software for further information.

Data

Policy information about availability of data

All manuscripts must include a data availability statement. This statement should provide the following information, where applicable:

- Accession codes, unique identifiers, or web links for publicly available datasets
- A description of any restrictions on data availability
- For clinical datasets or third party data, please ensure that the statement adheres to our policy

The RNA-seq, CUT&Tag and WGBS data have been deposited in the Gene Expression Omnibus (GEO) database. Accession number (GSE237800). Data are publicly

available upon publication. The source data for figures are provided as Supplementary Data 1. The uncropped images of genotyping gels are provided in Supplementary Information as supplementary Fig. 5.

Human research participants

Policy information about studies involving human research participants and Sex and Gender in Research.

Reporting on sex and gender	Study did not involve human research participants and human materials.
Population characteristics	Study did not involve human research participants and human materials.
Recruitment	Study did not involve human research participants and human materials.
Ethics oversight	Study did not involve human research participants and human materials.

Note that full information on the approval of the study protocol must also be provided in the manuscript.

Field-specific reporting

Please select the one below that is the best fit for your research. If you are not sure, read the appropriate sections before making your selection.

☒ Life sciences ☐ Behavioural & social sciences ☐ Ecological, evolutionary & environmental sciences

For a reference copy of the document with all sections, see nature.com/documents/nr-reporting-summary-flat.pdf

Life sciences study design

All studies must disclose on these points even when the disclosure is negative.

Sample size	Three independent ESC lines of each genotype is used for analyses in this study.
Data exclusions	No data were excluded from consideration.
Replication	Three independent ESC lines of each genotype is used for analyses in this study.
Randomization	Randomization was not required for this study
Blinding	Researchers were not blinded.

Reporting for specific materials, systems and methods

We require information from authors about some types of materials, experimental systems and methods used in many studies. Here, indicate whether each material, system or method listed is relevant to your study. If you are not sure if a list item applies to your research, read the appropriate section before selecting a response.

Materials & experimental systems

n/a	Involved in the study	
☐	☒	Antibodies
☐	☒	Eukaryotic cell lines
☒	☐	Palaeontology and archaeology
☒	☐	Animals and other organisms
☒	☐	Clinical data
☒	☐	Dual use research of concern

Methods

n/a	Involved in the study	
☒	☐	ChIP-seq
☒	☐	Flow cytometry
☒	☐	MRI-based neuroimaging

Antibodies

Antibodies used	Anti TET3 Rabbit Poly Clonal Antibody From Millipore (ABE290) listed and detailed in the methods and supplementary table 2.
Validation	The TET3 antibody is validated by the manufacturer Millipore (https://www.emdmillipore.com/US/en/product/Anti-TET3,MM_NF-ABE290?ReferrerURL=https%3A%2F%2Fwww.google.com%2F).

Eukaryotic cell lines

Policy information about cell lines and Sex and Gender in Research

Cell line source(s)	Tet3m/m (catalytic deficient) and Tet3–/– (knockout) mouse embryonic stem cell lines generated in the lab for this study.
Authentication	Genotypes of cell lines are validated by PCR, restriction fragment length polymorphism (RFLP) and Sanger sequencing.
Mycoplasma contamination	All cell lines have tested negative for mycoplasma.
Commonly misidentified lines (See ICLAC register)	No commonly misidentified cell lines were used in this study.
